# The Impact of Ecological Footprint, Urbanization, Education, Health Expenditure, and Industrialization on Child Mortality: Insights for Environment and Public Health in Eastern Europe

**DOI:** 10.3390/ijerph21101379

**Published:** 2024-10-18

**Authors:** Gheorghe H. Popescu, Elvira Nica, Tomas Kliestik, Cristina Alpopi, Ana-Madalina Potcovaru Bîgu, Sorin-Cristian Niță

**Affiliations:** 1 Department of Finance, Banking and Accounting, Faculty of Finance, Banking and Accounting, ”Dimitrie Cantemir” Christian University, 030134 Bucharest, Romania; popescu_ucdc@yahoo.com; 2 Doctoral School of Economics, Bucharest University of Economic Studies, 010374 Bucharest, Romania; 3 Department of Administration and Public Management, Faculty of Administration and Public Management, Bucharest University of Economic Studies, Piața Romană, 6, 010374 Bucharest, Romania; cristina.alpopi@ase.ro (C.A.); ana.bigu@amp.ase.ro (A.-M.P.B.); 4 Department of Economics, Faculty of Operation and Economics of Transport and Communications, University of Zilina, 010 26 Zilina, Slovakia; tomas.kliestik@fpedas.uniza.sk; 5 UNESCO Chair for Business Administration, Faculty of Business Administration in Foreign Languages, Bucharest University of Economic Studies, Piața Romană, 6, 010374 Bucharest, Romania; cristians.nita@fabiz.ase.ro

**Keywords:** child mortality, Eastern Europe, environment, health expenditure, ecological footprint, industrialization

## Abstract

The purpose of this study is to examine the connection between child mortality in Eastern Europe and ecological footprint, urbanization, education, health expenditure, and industrialization. The study acknowledges the significance of understanding how these factors influence the infant mortality rates in this region from 1993 to 2022. The Grossman Health Outcome (H-O) model investigates the theoretical framework. For the existence of the cross-sectional dependency, mixed-order unit root, and cointegration problem, the famous Cross-Sectional Autoregressive Distributed Lag (CS-ARDL) approach is applied. The research also used the Augmented Mean Group (AMG) and Common Correlated Effects Mean Group (CCEMG) to check robustness. The findings illustrated that health expenditure and education lessen the infant mortality rate in Eastern European countries. But ecological footprint, industrialization and unemployment raise the infant mortality rate. According to the CS-ARDL findings, expenditure on healthcare significantly reduces child mortality. Still, the ecological footprint significantly impacts increasing child mortality. However, the AMG and CCEMG models demonstrate that investing in education is the most effective strategy for reducing child mortality. Therefore, the government of Eastern European countries should provide more priorities in the sustainable urbanization, health expenditure, and education sectors. The robustness of the AMG and CCEMG also demonstrated the strength of the CS-ARDL findings. This research paper contributes to SDG 3 by examining the environmental and health factors that influence child mortality in Eastern Europe. Policymakers, public health professionals, and other stakeholders can use the findings to inform the development and implementation of programs that specifically target the identified causes of child mortality.

## 1. Introduction

Understanding the factors influencing child mortality in Eastern Europe is crucial for developing effective interventions to improve health outcomes. When socialist regimes in Eastern Europe collapsed at the end of the 1980s, the region changed dramatically as free markets were introduced [1]. While significant progress has been made in many areas over the past two decades, transitioning to a market economy and integrating with Western society still presents new challenges to these countries. It is important to remember that different Eastern European countries have diverse populations, incomes, development levels, and other social and financial aspects. Still, they share many of the same desires and difficulties [2]. Among the challenges, the under-5 mortality rate is one of the most significant problems. In developing countries, infant mortality is disproportionately high compared to its role in developed nations [3]. The rate of deaths among infants younger than one year old expressed as a percentage of total live births each year is known as the infant mortality rate [4]. In addition to its significance as a measure of child health, the IMR is also employed as a dependent or independent variable to assess a country’s level of socioeconomic development [5]. Eastern European healthcare systems underwent significant changes after the 1990s due to the political, social, and economic shifts that characterized these countries. The reforms have addressed all aspects of the health system, including funding, service delivery, management, and resource growth. Eastern European economies are diverse, as are their healthcare systems and their experiences with reform [6]. Considering all of this, this paper aims to investigate the causes of the disparities in health outcomes (as measured by the under-5 mortality rates) between Eastern European countries.

As a percentage of total births, the under-5 mortality rate indicates how likely it is that a child will not live to reach age 5. About 5,000,000 children under the age of 5 perished in 2021. With premature birth and intrapartum complications, infectious diseases such as pneumonia, diarrhea, and malaria account for a disproportionate share of deaths among children under five worldwide [7]. Fifty-nine percent has been cut from the global under-five mortality rate, from 93 deaths per 1000 live births in 1990 to just 38 deaths per 1000 in 2021. Although much progress has been made, there is still a pressing need to boost infant and child survival rates. About 13,800 children under five died daily in 2021, an intolerably high and largely preventable death toll [8]. IMR is a reasonable indicator that would be affected by many different characteristics overtly and covertly. Infants have a much lower chance of survival than adults and the elderly because their bodies are not fully developed, leaving them susceptible to all sorts of diseases and unable to adapt to new environments. This is why the under-5 mortality rate is so sensitive to changes in factors such as healthcare spending, carbon emissions, industrialization, urbanization, unemployment, and education.

In 2020, healthcare costs were projected to rise by 5% on average. Comparatively, private healthcare spending declined by more than 3 percent. Nevertheless, rising costs and slowing economies have led to a boost in health spending as a percentage of GDP in OECD countries to 9.7 percent in 2020 from 8.8 percent in 2019 [9]. In 2021, health spending was expected to increase by around 6%, according to preliminary estimates for 20 OECD countries. Despite this, the OECD Health Statistics [9] database projects that health spending as a share of GDP did not increase in 2021 as economies worldwide began to recover.

Greenhouse gas emissions (GHG) are rising globally, representing a significant risk to human beings and the environment [10]. According to the World Health Organization [11], between 2030 and 2050, climate change will be responsible for approximately 250,000 deaths per year. In addition, human health is negatively impacted by global warming and environmental degradation, both exacerbated by carbon emissions [12]. Many developing economies will inevitably pay the costs of environmental degradation as the energy and industrial sectors serve as primary drivers of economic development and growth. Therefore, for developing economies, the issue of linking human well-being to environmental degradation and economic progress is crucial, especially in those with weak regulatory environmental policies [13,14], inadequate regulatory infrastructure [15,16], and ineffective healthcare [17]. Instead of carbon dioxide and greenhouse gases, this study used ecological footprint. It is more comprehensive in its consideration of environmental impact than measures of carbon dioxide or other forms of pollution. The ecological footprint is the area of land and water needed to produce a given population’s worth of resources sustainably and to dispose of its waste. Not only does it factor in carbon dioxide emissions, but also how much food, water, and land are used for farming, logging, and building. The ecological footprint is a quantitative method of assessing the global effects of human activities on biodiversity and related ecosystem services. In contrast, carbon dioxide (CO_2_) and greenhouse gas (GHG) emissions are quantifications of the greenhouse gases released due to human activity and contributors to climate change. Burning fossil fuels for energy production and transportation, as well as industrial activities and changes in land use, are the primary sources of these emissions.

The health of newborns can suffer from, or benefit from, industrialization. Pollutants are released into the air and water by manufacturers. One of the leading causes of infant death is acute respiratory infections, and air pollution is a significant contributing factor [18]. By contrast, industrialization can boost health by enhancing residential happiness, hygiene, and pollution control. Similarly, improved maternal and infant nutrition and access to healthcare have been linked to declining mortality rates as per capita income has increased [19]. Several earlier studies concluded that children living in poor households with unemployed parents were at an increased risk of being neglected and abused physically [20,21]. Although some evidence suggests that assisting parents who cannot find work could be an efficient way to lessen the number of children harmed by their parents, implementing programs along these lines is prohibitively expensive. Lack of employment opportunities likely hurts birth outcomes because of maternal stress, increased alcohol consumption, and inadequate prenatal and postnatal care [22,23,24]. Unemployment negatively influences the number of young children who die before their fifth birthday.

**Figure 1:**
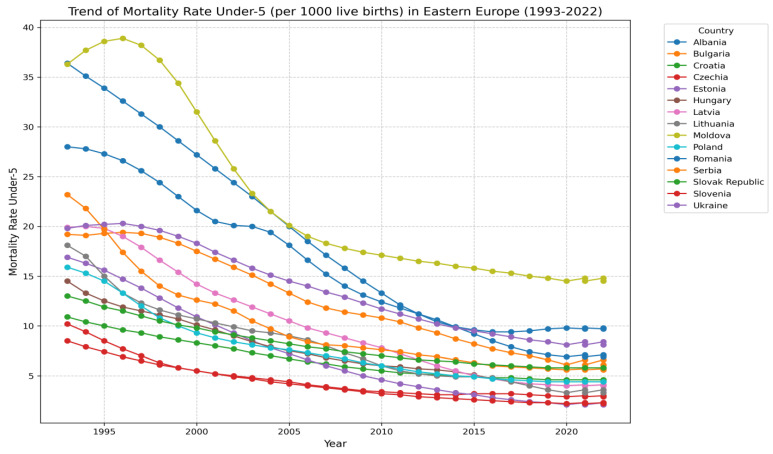
Trend of under-5 mortality rate in Eastern Europe (1993–2022).

Women’s educational attainment has a corrosive effect on the infant mortality rate [25,26]. This is because an educated mother will have better knowledge and awareness about health and disease prevention, allowing her to care for her child and herself during and after the pregnancy. This contrasts with an uneducated mother, who will have less of this knowledge and awareness. Therefore, it is recommended that mothers receive formal education to increase their understanding of health and wellness topics like healthy eating, infection prevention, and childbirth preparation [27]. Infant mortality correlates significantly with the mother’s education level [28,29,30]. Researchers in the social sciences agree that maternal education is a significant determinant of infant mortality [31,32,33]. The following Figure 1 illustrates the trend in under-5 mortality rates in Eastern Europe from 1993 to 2022, highlighting significant fluctuations over the decades. The data indicate a general decline in mortality rates, reflecting improvements in healthcare and socioeconomic conditions, although some periods show a plateau or slight increases due to regional crises and health disparities.

So, the objectives and contributions of this paper are multiple: 

(a) Eastern European countries were chosen for this study because they have higher child mortality rates than other developing and developed regions worldwide. On the other hand, they have undergone significant changes due to urbanization, industrialization, and environmental degradation, which may affect child mortality rates.

(b) We examine how environmental impact, urbanization, education, health spending, and industrialization affect Eastern European child mortality rates separately and together. All the factors were carefully chosen because of their significance to Eastern European countries. Environmental Footprint Variables were used in place of Carbon Dioxide and Greenhouse Gases. Eastern European countries are quickly urbanizing. Given this, it is essential to think about the study’s findings. The rate of industrialization is increasing in these nations. This was another factor considered in the study. Expenditures in the fields of health and education are rising in these nations. Both factors are considered throughout this study. 

(c) The CSD and slope homogeneity (SH) tests of the second generation were used in this study. The CSD and SH issues were mainly overlooked in earlier studies. Through trade, education, tourism, and other agreements, Eastern European countries are deeply interconnected. As a result, the data almost certainly suffer from cross-section dependency (CSD). 

(d) We analyze the panel data using the CS-ARDL, AMG, and CCEMG techniques, and measure the size and significance of the impacts of the factors on infant and child mortality rates. The three methodologies used for estimation are all second-generation panel tests. The CS-ARDL, AMG, and CCEMG are examples of second-generation panel tests that outperform their predecessors in both efficiency and power by providing greater flexibility and accounting for the possibility of cross-sectional dependence among the individual units in the panel.

The following hypotheses are formulated to explore the impact of various socio-economic and environmental factors on child mortality in Eastern Europe.

**H_1_.**  *Higher ecological footprint is positively associated with increased child mortality (lnMR) in Eastern Europe.*

**H_2_.**  *Increased urbanization has a positive effect on child mortality in Eastern Europe.*

**H_3_.**  *Greater current health expenditure is negatively associated with child mortality in Eastern Europe.*

**H_4_.**  *Higher industrialization contributes to an increase in child mortality in Eastern Europe.*

**H_5_.**  *Improved education levels are negatively associated with child mortality in Eastern Europe.*

**H_6_.**  *Higher unemployment rates are positively associated with child mortality in Eastern Europe.*

## 2. Literature Review

Understanding the factors influencing child mortality in Eastern Europe is essential for developing effective interventions to improve health outcomes and promote regional sustainability. Houeninvo [34] analyzed how healthcare spending influenced infant mortality rates in 37 African countries from 1995 to 2018. His research showed that higher public health spending was associated with lower death rates. It has been shown that when combined with spending on health, private health expenditures had a favorably impactful positive effect on the death rates of infants and children. Ayimaleh [35] analyzed how health expenditure impacted the infant mortality rates in Cameroon from 2000 to 2017. Maternal mortality and infant mortality were found to be negatively correlated with healthcare spending using ordinary least squares. Abbasi and Sohail [36] investigated how healthcare spending influenced the infant mortality rates in Southeastern Asian regions from 2000 to 2019. The GMM technique demonstrated that increased spending on medical care results in longer life expectancies and decreases in both crude and newborn mortality rates. Singh et al. [37] examined how public and private healthcare spending influenced the under-5 mortality rates. They found that increments in healthcare spending have been linked to longer life expectancy at birth, lesser rates of death for children under five, and lower rates of death from neurological illnesses. Gasimli et al. [38] examined the effects of energy intensity, carbon dioxide, and urbanization on infant mortality rates. Death rates were higher in areas where the environment had deteriorated.

The rates of infant mortality in Asia grew from 1991 to 2019, according to research by Zhang et al. [39], even though increments in GDP, healthcare costs, and urban population worked to reduce the rate. Child mortality rates were studied by Dhrifi [40] from 1995 to 2012 in 93 different developed and developing countries. He accounted for the effects of healthcare expenditures, environmental damage, GDP, and urbanization. Researchers found that health spending reduces infant mortality only in countries with high per capita incomes. In contrast to urbanization, which reduces mortality, and ecological footprint, which increases mortality, GDP had a sizeable impact on decreasing child mortality. Raises in carbon dioxide emissions and child mortality in Sub-Saharan Africa were reported by Onanuga et al. [41]. At the same time, economic growth negatively affected the infant mortality rate, and a growing urban population and high fertility had a highly positive effect. Senol et al. [42] analyzed how health expenditures, GDP, and unemployment impacted the under-5 mortality rates in 132 countries from 2000 to 2019. Following the conclusions, a rise in unemployment correlated with a higher infant mortality rate. In contrast, a boost in public spending was inversely associated with decreased infant mortality. Oikawa et al. [43] found that the unemployment rate enhanced the infant mortality rates in Japan from 2005 to 2016.

Sepehrdoust et al. [44] analyzed how human development indicators impacted the infant mortality rates in Iran from 1987 to 2017. They concluded that the unemployment rate boosted the under-5 mortality rates. Nguyen-Phung [45] analyzed how maternal education impacted the infant mortality rates in Vietnam from 1997 to 2002. He found that increasing maternal education alleviated infant mortality rates. Rahman and Alam [46] analyzed how urbanization and female education impacted the child mortality rates in Bangladesh from 1975 to 2019. The ARDL model illustrated that urbanization enhanced child mortality, but education decreased it. Safdar et al. [47] analyzed how industrialization and urbanization impacted the infant mortality rates in South Asian countries from 2000 to 2020. The PMG-ARDL method demonstrated that industrialization accelerated infant mortality rates, but urbanization decreased the mortality level. Finally, Sule et al. [48] analyzed the influence of education and energy on the under-5 mortality rates in 35 African countries. The FMOLS method illustrated those educational advantages alleviated child mortality rates.

Furthermore, limited attention has been paid to the combined effects of multiple risk factors on child mortality. A systematic review by Wang [49] emphasizes the necessity of understanding how various socio-economic determinants interact to influence health outcomes, advocating for a multi-dimensional approach to child mortality studies. This is echoed by the findings of Kennedy et al. [50], which highlight that combinations of high unemployment and low educational attainment are particularly detrimental in specific regional contexts. By incorporating these nuanced insights into our analysis, we aim to not only clarify the individual impacts of factors such as ecological degradation, health expenditure, and urbanization but also explore how their interactions contribute to child mortality in Eastern Europe. For instance, a meta-analysis by Qiu et al. [51] demonstrated that while individual factors like health expenditure and educational attainment have significant impacts, their combined effects can be more pronounced. The authors emphasize that the interaction between low educational levels and inadequate health services can synergistically increase child mortality rates, particularly in low- and middle-income countries. Moreover, research by Soares [52] highlighted the importance of contextual factors, noting that the effects of socio-economic determinants on health outcomes vary considerably across different regions. This variability underscores the need for more localized studies that examine how specific combinations of risk factors, such as urbanization and environmental degradation, contribute to child mortality. A study by Li et al. [53] examined how combinations of factors like poverty, education, and access to healthcare create a cumulative risk effect on child mortality. They found that children living in households that fall below a specific income threshold while simultaneously lacking access to quality healthcare have significantly higher mortality risks. Their findings suggest that policymakers should consider the thresholds of these interacting factors when devising strategies to reduce child mortality.

According to reviewed researchers, no study has examined the impact of health expenditure, pollution, urbanization, industrialization, education, and unemployment on child mortality in the nations of Eastern Europe combined. Greenhouse gases and carbon dioxide emissions have been used in most previous studies as a stand-in for pollution. This research uses the ecological footprint concept to quantify environmental damage and contamination. When assessing environmental health, the ecological footprint is a more robust metric than carbon dioxide and greenhouse gas emissions. Previous researchers in other regions also used traditional first-generation regressions like SGMM, ARDL, FMOLS, and DOLS. There are limitations to these econometric approaches, though. The countries of Eastern Europe are also very intertwined with one another because of trade, education, tourism, and other agreements. As a result, the data almost certainly suffer from cross-section dependency (CSD). Most prior studies glossed over the issue of CSD. 

This study tries to address this gap by employing the CS-ARDL method after confirming the CSD, the slope homogeneity problem, and the mixed-order stationary problem. The AMG and CCEMG estimators were used to test for stability. All three estimating methods use panel tests of the second generation. The CS-ARDL AMG and CCEMG are examples of second-generation panel tests that outperform their predecessors in both efficiency and power by providing greater flexibility and accounting for the possibility of cross-sectional dependence among the individual units in the panel. Conventional panel models treat each team in the panel as unique. The units may be interdependent on one another cross-sectionally due to shared shocks or spillover effects. The CS-ARDL AMG and CCEMG assessments are more reliable since they consider this cross-sectional dependency. The CS-ARDL AMG and CCEMG tests have outperformed conventional panel models regarding power and size distortion. This improves their ability to identify genuine connections between the relevant variables. The CS-ARDL AMG and CCEMG tests can estimate panel data models more effectively. They do this by capitalizing on the panel units’ cross-sectional dependence and common factors. Compared to conventional panel models, the CS-ARDL AMG and CCEMG tests are more resistant to insufficient data. This is paramount when conducting empirical investigations with small sample sizes.

## 3. Methodology

### 3.1. Theoretical Framework and Health Production Function

The effects of healthcare spending, carbon dioxide emissions, industrialization, urbanization, and lack of education on infant mortality are examined. Since the introduction and literature review sections analyzed the impact of the dependent and independent variables, the HO model developed by Grossman [54] has been chosen as the theoretical framework for this study. Grossman [54] presumed health to be a form of capital and employed valuation models to investigate the relationship between health and factors like age, income, and education level, among others, as well as regarding the health endowment. This method analyzes health as a stock of resources that undergoes constant fluctuation, much like depreciable capital. Conditions like illness and advancing age can deplete one’s health reserves. There are two main drivers behind the demand for healthcare in modern society. For starters, let us look at healthcare as a consumption good, the source of whose disutility is lost workdays. Second, it can be seen as a financial asset that influences how one prioritizes market and non-market activities [54]. The maximum possible output for a given input is depicted by the classical production function(s). According to the health production function model, a person’s health is affected by several factors, some of which are under his control.

The Grossman [54] model predicts that a person’s health will be affected by their health stock, healthcare service utilization, and several other variables, as shown in Equation (1).

(1) $$Health=\int \left(\alpha ,D,X\right)$$

where α stands for an individual’s baseline health stock, which is affected by genetics and social capital, *D* stands for an individual’s demand for healthcare services, and *X* stands for a vector of other factors, including economic and social aspects.

Although this theoretical model was conceived to examine health output at the micro level, its applicability to the macro level remains intact [55,56]. This is because the economic, social, environmental, and health service utilization variables that make up the health production inputs at the macro level are most shown in the form of their per capita values, as shown in Equation (2).

(2) $$Health=\int \left(Y,S,E\right)$$

In Equation (3), three vectors represent everyone’s economic, social, and environmental conditions: *Y*, *S*, and *E*, correspondingly.

(3) $$Health=\int \left(HEX,EF,INDUS,URBA,EDU,UNEM\right)$$

In this study, the paper defines health expenditures (*HEX*) as an economic component, urban population (*URBA*), industrialization (*INDUS*), and ecological footprint (*EF*) as environmental variables, and education (*EDU*) and unemployment (*UNEM*) as social factors.

The under-5 mortality rate can be broken down into its contributing factors by using the following Equation (4) as a guide:

(4) $$MR_{it}=\alpha_{0}+\alpha_{1}HEX_{it}+\alpha_{2}{EF}_{it}+\alpha_{3}INDUS_{it}+\alpha_{4}URBA_{it}+\alpha_{5}{EDU}_{it}+\alpha_{6}UNEM_{it}+\varepsilon_{it}$$

where *MR* stands for the infant mortality rate and is our dependent variable of interest for this analysis, and *HEX*, *EF*, *INDUS*, *URBA*, *EDU*, and *UNEM* are explanatory variables on the right. They each reflect the state of healthcare spending, ecological footprint, industrialization, urbanization, education, and unemployment.

This publication followed the work of Majeed and Ozturk [57] and Muhammad and Siddique [58] by computing the logarithm of the variables.

(5) $$lnMR_{it}=\alpha_{0}+\alpha_{1}lnHEX_{it}+\alpha_{2}{lnEF}_{it}+\alpha_{3}lnINDUS_{it}+\alpha_{4}lnURBA_{it}+\alpha_{5}{lnEDU}_{it}+\alpha_{6}lnUNEM_{it}+\varepsilon_{it}$$

Micro-level studies were undertaken by researchers such as Grossman [54], Balia and Jones [59], Khuder [60], Wise [61], Nixon and Ulmann [62], and Pedrick [63]. The primary advantage of this approach is that it allows for the prediction of the full impact of health spending and other factors unique to the health of a community. Research into health outcomes can benefit from using the framework provided by the Grossman model of health production. The benefits of the Grossman [54] theory have attracted the attention of many researchers, including Bayati et al. [64], Azam et al. [65], Miller and Frech [66], Tariq and Xu [67], Cremieux et al. [68], Xu et al. [69], Lei et al. [70], Rodgers [71], Auster et al. [72], and Popescu [73].

### 3.2. Data

Table 1 shows that all variables are collected from the world development indicators (WDIs). Here, the under-5 mortality rate is the dependent variable, and electricity, ecological footprint, GDP, industrialization, urbanization, and fertility rate are the explanatory variables of this study. The duration of data is 1993 to 2022. There are 15 countries listed here. Appendix A provides a comprehensive list of the Eastern European countries examined in this study. Also, Appendix B lists the abbreviations used for key variables and terms in the study. In this research, Excel was utilized for data cleaning and preparation, Stata was employed for conducting regression analyses, and Python was used to generate visual representations and figures. The research utilized extrapolation and interpolation techniques to address and fill in missing values in the dataset.

Table 2 summarizes descriptive data for seven variables (lnMR, lnHEX, lnEF, lnURBA, lnINDUS, lnEDU, and lnUNEM). The number of measurements (N) for each variable is shown in Table 1. The mean value of each variable is given to provide insight into the data’s primary trend. Standard deviations are provided for each measure to show how widely the data can range. The minimum and maximum numbers for each variable display the observed range. Overall, this chart gives a quick overview of critical statistical measures for these variables, which can help gain insight into their distribution and characteristics.

### 3.3. Econometric Framework

In this research examining the connection between child mortality in Eastern Europe and variables, a robust econometric framework is essential to ensure reliable findings. The initial step involved applying a slope homogeneity test, which assesses whether the relationships between the dependent and independent variables are consistent across different countries. Following this, a CSD test was conducted to determine the extent of interdependence among the panel data. Given the potential for cross-sectional dependency in economic data, this step is critical, as it informs the choice of econometric techniques that can handle such complexities effectively.

After confirming the presence of cross-sectional dependency, the research employed the second-generation unit root test, specifically the CIPS test, to assess the stationarity of the variables. This is important, as non-stationary data can lead to spurious results. The presence of mixed-order unit roots was established, indicating that different variables might require differencing at different orders. Subsequently, the Westerlund cointegration test was utilized to investigate long-term relationships among the variables, ensuring that the econometric model captures not just short-term dynamics but also enduring associations between child mortality and its determinants. Finally, the research employed the CS-ARDL model, which accommodates both short-run and long-run relationships while controlling for cross-sectional dependence. To enhance the robustness of the findings, the study also incorporated the AMG and CCEMG estimators. These methods provide further verification of the results obtained from the CS-ARDL analysis, accounting for potential biases introduced by unobserved heterogeneity and cross-sectional dependence. 

## 4. Results and Findings

**Figure 2:**
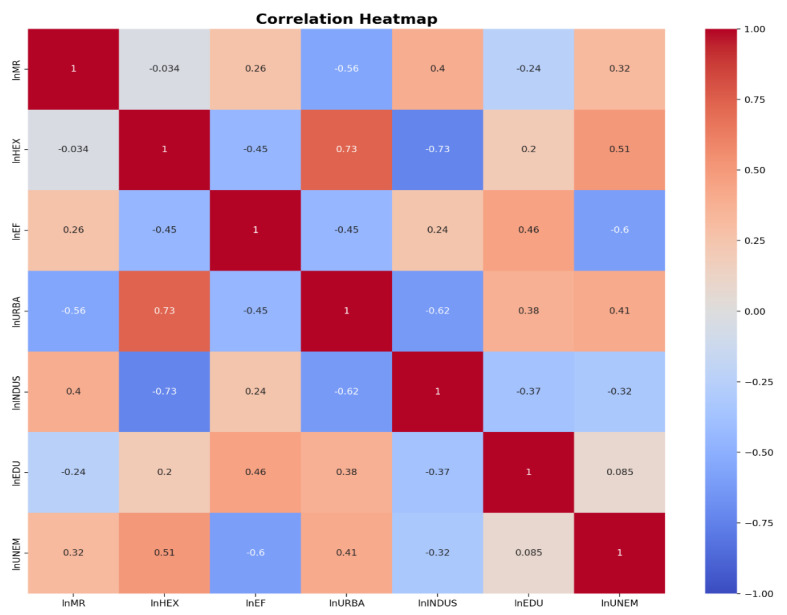
Correlation Heatmap of Socioeconomic and Health Indicators in Eastern Europe.

The following Figure 2 presents a correlation heatmap of various socioeconomic and health indicators in Eastern Europe, indicating that no correlations exceed 0.80, suggesting a moderate degree of independence among the factors. This visualization allows for the identification of potential relationships that may influence child mortality while maintaining clarity regarding the distinct impacts of each indicator.

The results of a test performed by Pesaran and Yamagata [76] to establish whether slopes were consistent across the sample are displayed in Table 3. The model has a heterogeneity issue, according to the findings. The results show that the parameters in the model are not constant and that the slope varies across countries. If the premise of a continuous slope is considered, panel causal analysis may produce correct results by imposing a homogeneity restriction on the dependent variable.

**Figure 3:**
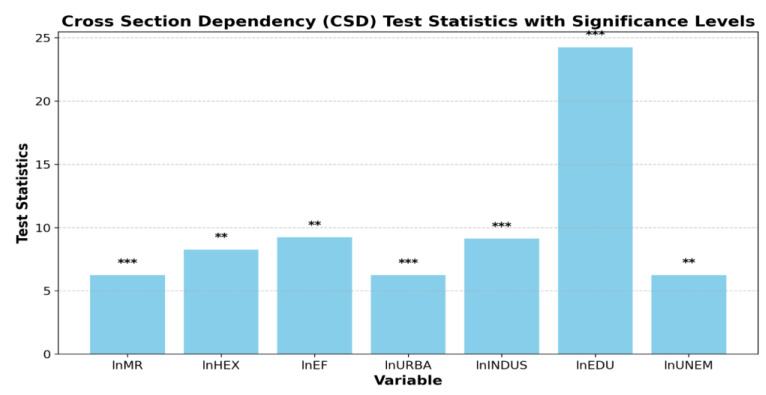
CSD statistics value with significant levels.

Figure 3 demonstrates that the test statistics score for the chosen independent and dependent variables has shown highly significant values with a significance level of 1% and 5% (** and ***). This indicates that CSD is present in the research data, and the alternative hypothesis is accepted at a 1% significance level.

According to the previous discussion, the CS-ARDL method is perfect regardless of whether the variables are I(0), I(1), or a combination. Hence, it is essential to pay attention to the stationarity characteristics of our variables. However, according to some studies (De V. Cavalcanti et al. [77]; Eberhardt and Presbitero [78]), the existence of cross-sectional dependence threatens the validity of the standard panel unit root test. For this reason, we used the Cross-Sectional Augmented Im–Pesaran–Shin (CIPS) test in Table 4 (Pesaran [79]). By adding cross means to the ADF regression for each unit, this test (CIPS) accommodates the heterogeneous unit process. The unit root test results demonstrate the mixed-order stationary nature of all factors.

Additionally, the cointegration test based on the Persyn and Westerlund [80] examination is displayed in Table 5. The results are also evidence of cointegration between the model factors.

This study uses various measures in the earlier stages, including CSD, slope heterogeneity, unit root, and panel cointegration. Then, using CS-ARDL, we present the empirical results for both the long term and the short term.

Table 6 demonstrates the conclusions of the CS-ARDL model findings. The health expenditure significantly alleviated the infant mortality rates in Eastern Europe. A 1% increase boosts healthcare spending significantly, causing a 0.556% decline in mortality rates. Abbasi and Sohail [81] in the Southeastern Asian region demonstrated that healthcare spending alleviated infant mortality rates. By contrast, ecological degradation badly influences infants’ health in Eastern Europe. It raises the death rates among infants. Thus, 1% enhances the ecological footprint level, inducing a significant 0.0845% rise in infant mortality rates. Several studies, including those by Voumik and Sultana [82], found that carbon emissions accelerated the mortality rate of infants. But the influence of the urban population on infant mortality rates is beneficial, as it lowers the infant death rates. If the urban population rises by 1%, then mortality rates decline by 0.528%. Industrialization enhances infant mortality rates. A 1% increase boosts the industrialization growth significantly, suggesting a 0.0648% rise in the infant mortality rates in Eastern Europe. In Indonesia, Federman and Levine [83] demonstrated that industrialization increased child mortality rates. By contrast, education contributes a crucial role in reducing infant mortality rates. Education alleviates the mortality rates by 0.110% through a 1% rise in education. Rahman and Alam [46] in Bangladesh, Asif et al. [84] in Pakistan, Kiross et al. [85] in Ethiopia, and Balaj et al. [86] demonstrated that education mitigated infant mortality rates. Unemployment boosts the under-5 mortality rates by raising the death rates. A 1% rise in the unemployment level significantly causes a 0.01716% boost in mortality rates. Researchers in Argentina [87], Asia [88], the United States [89], and 127 countries [90] showed that unemployment had a positive effect on the infant mortality rate. Healthcare spending, ecological footprint, industrialization, and unemployment significantly influence the under-5 mortality rates.

The short-run results of the CS-ARDL demonstrate that healthcare spending, urbanization, and education alleviate infant mortality rates. A 1% enhancement in the healthcare spending, urbanization, and education induces 1.720%, 0.035, and 0.0117% alleviation of the infant mortality rates, respectively. Ecological footprint, industrialization, and unemployment enhance the under-5 mortality rates. A 1% boost in the environmental footprint, industrialization, and unemployment causes a 0.03113%, 0.2785%, and 0.0045% rise in the infant mortality rates. A strong and significant long-run link between the variables is shown by an error correction value of −0.911, which shows that the model’s variables are co-integrated and move towards equilibrium at 91.1% in each period.

Table 7 displays the outcomes of a regression study that used the AMG and CCEMG techniques to examine the interplay between many factors and infant mortality in Eastern European countries. The computed coefficients and standard errors for the AMG and CCEMG models are presented in columns 2 and 3, respectively. Estimates for the coefficient of lnHEX show a negative and significant relationship between health spending and infant mortality in Eastern European nations, with a negative value of −0.0419 (AMG) and −0.0700 (CCEMG). There is statistical evidence from the coefficients, at both the 10% and 5% levels, indicating that healthcare spending correlates with lower infant and child mortality rates. A bigger ecological footprint is linked to a higher child mortality rate, as noted in the results in both models, suggesting that lnEF has a significant positive effect on child mortality. Estimates for the coefficient of lnEF place the increase in child mortality rates in Eastern European nations at 0.0328% (AMG) and 0.0567% (CCEMG) for every 1% increase in ecological footprint. 

Higher degrees of urbanization are linked to reduced child mortality rates, whereas lnURBA substantially affects child mortality in the CCEMG model. For Eastern European nations, a 1% rise in urbanization is linked to a 0.0855% to 0.3% drop in child mortality rates (lnURBA = −0.0855 for AMG and −0.300 for CCEMG). A 1% increase in industrialization relates to a 0.00391–0.00519% increase in child mortality rates in Eastern European nations, according to the calculated coefficient of lnINDUS, which is 0.00391 * (AMG) and −0.00519 (CCEMG). In the AMG approach, the coefficient is significant at the 10% level, while in the CCEMG method, it is not. Although the effect is inconsistent between the two estimating methods, this finding suggests a little beneficial and significant influence of industrialization on child mortality rates in the region. With an estimated coefficient of lnEDU of −0.00848 (AMG) and −0.0335 (CCEMG), we learn that every point gained in educational attainment is linked to a drop in infant mortality of 0.00848% to 0.0335% across Eastern European nations. Both models’ lnUNEM coefficients are non-significant, indicating that fluctuations in Eastern European unemployment do not affect child mortality rates. Most of our AMG and CCEMG findings are consistent with those of the CS-ARDL. In most instances, even though the magnitudes differ, the sign and direction remain the same.

## 5. Discussion

Findings show that increasing health expenditure minimizes child mortality. It is possible that the accessibility and availability of healthcare services could improve if the health expenditure and budget were raised. Examples are hiring more medical professionals, developing new healthcare facilities, and distributing new medical tools and apparatuses. Better health outcomes and lower child mortality rates can be achieved through increased access to healthcare, which can help guarantee that children receive treatment when needed. Prevention efforts like immunizations, food assistance, and health instruction can receive more funding and attention with a larger healthcare budget. An early diagnosis and course of treatment for a sickness can greatly lessen the likelihood of serious complications or death from that illness being untreated. Overall, better healthcare access, preventive care, illness treatment, and maternal health can contribute to lower infant mortality rates if healthcare spending and budgets are raised. 

Child mortality rates may be significantly affected by ecological footprint and pollution. Asthma, pneumonia, and bronchitis are just a few respiratory illnesses that can be triggered by prolonged exposure to high amounts of air pollution. Babies and toddlers are especially vulnerable because their breathing systems are still maturing. Low birth weight, premature delivery, and delayed development in children are all symptoms of exposure to high levels of air pollution. Children’s health is especially vulnerable to the long-term impacts of environmental pollutants like lead, mercury, and pesticides, including cognitive and developmental delays and an increased chance of chronic diseases like cancer. Lowering ecological footprint and pollution levels is essential to promote healthy environments for children, avoid respiratory illnesses, and improve overall health outcomes. Advances in computer-aided ecological footprint estimations provide valuable insights that can be integrated into future policies and health planning. By using such data, eastern European countries and health institutions can better understand environmental impacts and design interventions aimed at reducing childhood diseases, particularly in regions experiencing rapid urbanization and industrial growth. 

It has been shown that urbanization and access to urban infrastructure can play an important part in reducing child mortality rates. Greater concentrations of hospitals, clinics, and specialized medical services can be found in urban regions compared to their more rural counterparts. By getting people the help they need sooner, we can lessen the likelihood of complications and mortality from diseases and accidents. Diarrheal diseases are a major contributor to the premature death of children worldwide. Still, they may be prevented or treated more effectively in urban regions due to improved access to clean water, sanitation systems, and hygiene facilities. Additionally, the transportation systems and infrastructure in metropolitan areas are typically superior, making reaching medical facilities and emergency services simpler in times of need. Children’s overall health and access to urban amenities can improve, reducing child mortality rates. 

Industrialization can have a negative impact on child mortality rates in several ways. The rapid expansion of industries can lead to increased air, water, and soil pollution, which can have adverse health effects on children. High pollution levels can lead to respiratory illnesses, such as asthma, bronchitis, and pneumonia, which can be particularly dangerous for young children whose respiratory systems are still developing. Industrialization can also lead to poor working conditions and occupational hazards, which can affect the health of parents and caregivers and indirectly impact children’s health. Furthermore, industrialization can lead to urbanization and the growth of urban slums, which often lack access to basic services such as clean water, sanitation systems, and healthcare facilities, putting children at increased risk of infectious diseases and other health problems. Therefore, it is essential to ensure that industrialization is carried out sustainably and responsibly, with appropriate measures to protect children’s and their family’s health. 

An important factor in reducing infant mortality is access to quality education. Better health results can be achieved for children if more people know the significance of healthcare, nutrition, and hygiene practices. Mothers with higher levels of education are more likely to receive prenatal care, have hospital births, and breastfeed their infants, all of which benefit their children’s health [91]. Children’s health can benefit from increased access to healthcare and improved living circumstances made possible through increased family income, both of which are facilitated by education. In addition, by empowering women and fostering other social and cultural shifts, education can enhance children’s health and nutrition. Consequently, reducing infant mortality and improving community health largely depends on spending on education, especially for girls and women. 

Unemployment has been linked to increased infant mortality in several ways [92,93,94]. When parents are out of work, they may be unable to put food on the table or provide adequate housing for their children, which can negatively affect their health. Parents who are out of work may be under additional stress, which can negatively affect their well-being and that of their offspring. Unemployed people may also be less likely to have access to medical treatment because they lack the financial resources or health insurance to pay for necessary procedures. In addition, children are at a higher risk of being injured or killed because of the breakdown of social support networks and increased crime and violence that can result from unemployment. Child mortality rates can be significantly lowered, and the health of children and their families can be improved by addressing unemployment and fostering economic security.

## 6. Conclusions

In conclusion, addressing the issue of child mortality in Eastern Europe due to pollution, urbanization, education, health spending, and industrialization is a complex issue that calls for a multifaceted approach. Also, the nations of Eastern Europe are very intertwined with one another because of trade, education, tourism, and other agreements. Therefore, CSD is a severe issue likely present in the data. The Westerlund exam and a newer version of the CSD test were used in the study. This study aims to meet this need by employing the CS-ARDL method after confirming the CSD, slope homogeneity problem, and mixed-order stationary problem. The AMG and CCEMG estimators were used to test for stability. 

The CS-ARDL findings show that increasing ecological footprint, unemployment, and rapid industrialization are responsible for child mortality. On the other hand, health expenditure and education are minimizing the child mortality rate. The impact of urbanization is significant in the short run but insignificant in the long run. The CS-ARDL data show that healthcare spending significantly lowers child mortality, with a coefficient of −0.556 ***. At the same time, the ecological footprint has the highest impact on raising child mortality, with a coefficient of 0.0845 **. Coefficients for AMG and CMG models indicating that investing in education is the most effective strategy for reducing child mortality are −0.0848 ** and −0.0635 **, respectively. There is a strong and significant long-run link between the variables in the model, as indicated by an error correction value of −0.911 ***, which shows that the variables are co-integrated and tend to converge to equilibrium at a rate of 91.1% each period. 

So, reducing the ecological footprint, expanding access to urban services, funding education and healthcare, and encouraging environmentally responsible industrialization can reduce infant mortality rates. To ensure that children receive the care and support they need to flourish, lawmakers, healthcare providers, educators, and community leaders must work together to address these interrelated factors. Healthier, more sustainable, and more equitable societies for future generations can be achieved by prioritizing children’s health and well-being today.

## 7. Policy Recommendation

Increased health spending and prioritizing expenditures in healthcare systems are necessary for governments to reduce infant and child mortality rates. Several policy suggestions, such as boosting public spending on healthcare, expanding access to care for underserved populations, emphasizing the importance of preventative care, training a larger healthcare workforce, and partnering with foreign organizations, can help get us there. Governments can protect the health of all children, especially the most marginalized, by enacting these laws. Furthermore, these policies can aid in disease prevention, improving children’s health results and boosting community wellness [95,96,97]. In the long run, spending money on healthcare is the right thing to do ethically and economically because a healthy kid is more likely to develop into a healthy and productive adult.

The following are policy recommendations to reduce ecological footprints and defend the health of children. Sustainability initiatives, including green construction, renewable energy, and environmentally responsible farming, should be supported by the government. Incentives like tax deductions, subsidies, and grants can help accomplish this. Mandatory recycling, composting, and waste reduction targets are all examples of policies that governments might undertake to promote waste reduction. In addition, governments should encourage less reliance on personal automobiles by investing in public transit infrastructure. Governments may preserve children’s health and the environment by adopting these policies. These actions will secure a sustainable future for future generations and those living today.

Policymakers should emphasize environmentally responsible urbanization plans to reduce infant mortality rates. This can be accomplished through a variety of policy suggestions, including but not limited to advocating for the increased use of green infrastructure like parks and green spaces, enhancing public transportation, promoting sustainable building practices, encouraging energy efficiency and renewable energy sources, and investing in water and sanitation infrastructure [98,99,100]. Safer and healthier urban neighborhoods, lower pollution, and more physical exercise opportunities are all ways these policies can benefit city dwellers of all ages, especially children. In addition, green and sustainable urbanization can also help reduce greenhouse gas emissions and prepare communities for the consequences of climate change, such as more frequent and severe weather. Ultimately, investment in green and sustainable urbanization is not just a moral but also an economic necessity, as healthy and sustainable cities can lead to better economic growth and social development.

Industrialization has contributed significantly to Europe’s economic growth and development but has also been linked to detrimental health effects, including higher infant mortality rates [101,102,103,104]. The following policy suggestions can be considered to advance industrialization while enhancing health conditions and lowering infant mortality rates. Improving workplace safety regulations is one of the most important methods to reduce child mortality rates in industrialized nations. This can be accomplished by creating and enforcing safety laws, such as handling hazardous materials, machinery safety, and adequate worker training. Investing in the healthcare sector’s infrastructure can guarantee employees’ families access to quality medical treatment, including prenatal care and child immunizations. Creating environmental laws, such as those governing the quality of the air and water, can aid in reducing the harmful effects of industrialization on health. Promoting healthy lifestyles through programs like workplace wellness initiatives, access to recreational facilities, and healthy food choices can help to improve general health and lower child mortality rates. Finally, the management of waste can both enhance the environment and lower the death rate of children [105].

Governments should prioritize investments in education and education funds to reduce child mortality rates [106,107,108]. Several policy suggestions have been proposed to accomplish this goal, such as increasing public spending on education, expanding educational opportunities for all children, especially those from disadvantaged backgrounds, bolstering health education in schools, and funding teacher professional development. The best way to ensure that our children have the tools they need to grow healthy and prosperous is to prioritize education. In addition, children’s general growth and future achievements can be aided by their ability to make educated choices about their health and well-being, another benefit of education. More remarkable economic growth, lower poverty rates, and greater gender equality are just a few of the broader social and economic benefits of investing in education. If we want children to grow up to be healthy and productive members of society who add to the well-being of their communities, we must spend on their education and the budgets dedicated to it.

Child mortality rates are one element of society that unemployment can negatively impact. The following suggestions for policy can be considered to reduce unemployment in Europe and deal with this problem. The skills and employability of people can be increased by investing in education and training, which can result in more job opportunities and eventually lower unemployment rates [109,110,111]. Promoting entrepreneurship can result in the establishment of new companies and employment possibilities. Initiatives like tax breaks for new businesses, streamlined regulatory frameworks, and easier access to start-up funding can accomplish this. The building, engineering, and transportation industries, among others, can all benefit from investing in infrastructure. This can be accomplished by carrying out public works projects, investing in renewable energy sources, and enhancing transit infrastructure.

## 8. Limitations and Future Research

This study faces several limitations that should be acknowledged. Firstly, the analysis is constrained by the availability of consistent data across all Eastern European countries from 1994 to 2023, which may impact the robustness of the findings. Additionally, the selection of specific socio-economic and environmental variables, while critical, may overlook other influential factors such as healthcare quality, social protection policies, technological improvements, and political stability, which could also play significant roles in child mortality rates. Moreover, the methodological approach, though rigorous, primarily focuses on linear relationships, which may not fully capture the complexities or potential endogeneity among the variables being studied.

Future research could address these limitations by exploring non-linear dynamics between the variables to uncover more complex interactions affecting child mortality. Additionally, expanding the scope of the analysis to include factors such as healthcare quality, social protection policies, and environmental regulations would provide a more comprehensive understanding of the determinants of child mortality. Comparative studies that examine these relationships across different regions or country groups could also yield valuable insights, helping to determine whether the findings in Eastern Europe are specific to this region or have broader applicability.

## Figures and Tables

**Table 1:** Detailed list of variables with sources and logarithmic transformations for analysis.

Details	Short Form	Log Form	Source
Mortality rate under-5 (per 1000 live births)	MR	lnMR	World Development Indicator, WDI [74]
Current health expenditure (Current USD)	HEX	lnHEX	
Industry (including construction), value added (constant 2015 USD)	INDUS	lnINDUS	
Urban population (% of the total population)	URBA	lnURBA	
Education	EDU	lnEDU	
Unemployment, total (% of the total labor force) (modeled ILO estimate)	UNEM	lnUNEM	
Ecological Footprint (Global hectare of land)	EF	lnEF	Global Footprint Network [75]

**Table 2:** Summary statistics of key socioeconomic and environmental variables.

Variables	N	Mean	sd	Min	Max
lnMR	450	2.259	0.639	0.742	3.714
lnHEX	450	6.184	0.895	3.062	7.705
lnEF	450	1.854	0.321	1.124	2.654
lnURBA	450	4.072	0.173	3.595	4.331
lnINDUS	450	23.03	1.322	20.02	25.85
lnEDU	450	1.519	0.222	0.802	2.292
lnUNEM	450	2.293	0.586	0.0953	3.438

**Table 3:** Results of slope homogeneity tests using Δ and adjusted Δ statistics.

Slope Homogeneity Tests	Δ Statistic	*p*-Value
$\overset{ˇ}{\Delta }$ test	7.250 ***	0.001
${\overset{ˇ}{\Delta }}_{adj}$ test	8.356 ***	0.002

The foundation for a measure of slope heterogeneity is the presumption that all slope coefficients are homogeneous. The *** symbol indicates that less than 1% of the populace is represented.

**Table 4:** Unit root test results at level and first difference for variables.

Variables (in the log)	Level		First Difference		Order
	Without Trend	With Trend	Without Trend	With Trend	
lnMR	−0.824	−1.797	−2.639 ***	−3.014 ***	I(1)
lnHEX	−1.242	−2.009 *	−3.574 ***	−3.706 ***	I(1)
lnEF	−2.423 ***	−2.547 ***	−3024 ***	−3.293 ***	I(0)
lnURBA	−0.925	−1.026	−4.568 ***	−5.900 ***	I(1)
lnINDUS	−1.124	−1.774	−3.842 ***	−4.038 ***	I(1)
lnEDU	−2.181 **	−2.438 ***	−5.120 ***	−5.232 ***	I(0)
lnUNEM	−1.25	−2.256	−3.25	−4.025	I(1)

Significance levels are: *** *p* < 0.01, ** *p* < 0.05, * *p* < 0.1.

**Table 5:** Results of Westerlund panel cointegration tests for long-term relationships.

Statistic	Value	Z-Value	*p*-Value
Gt	−3.452 ***	−1.842	0.010
Ga	−6.539 ***	2.291	0.050
Pt	−4.475 ***	2.685	0.550
Pa	−3.886 ***	2.799	0.750

Here, *** *p* < 0.01.

**Table 6:** Results of CS-ARDL estimation: long-run and short-run dynamics.

Variables	Long-Run Results	Short-Run Results
lnHEX	−0.556 *** (0.077)	−1.720 *** (0.049)
lnEF	0.085 ** (0.033)	0.031 * (0.024)
lnURBA	−0.528 (0.591)	−0.035 ** (0.014)
lnINDUS	0.065 ** (0.027)	0.278 ** (0.137)
lnEDU	−0.110 * (0.066)	−0.012 (0.023)
lnUNEM	0.017 ** (0.084)	0.005 (0.024)
Adjusted Term	−0.911 *** (0.115)	
Number of countries	15	
R-squared	0.744	

Standard errors in parentheses; *** *p* < 0.01, ** *p* < 0.05, * *p* < 0.1.

**Table 7:** Estimation results from AMG and CCEMG methods (robustness check).

Variables	AMG	CCEMG
lnHEX	−0.042 * (0.022)	−0.070 ** (0.032)
lnEF	0.033 *** (0.034)	0.056 ** (0.038)
lnURBA	−0.085 (0.123)	−0.300 *** (0.055)
lnINDUS	0.004 * (0.032)	−0.005 (0.044)
lnEDU	−0.008 ** (0.003)	−0.033 ** (0.014)
lnUNEM	0.007 (0.010)	0.022 (0.036)
Constant	−1.271 (8.717)	7.008 (21.510)
Adj R^2^	0.924	0.965
Observations	450	450
Number of Countries	15	

Standard errors in parentheses; *** *p* < 0.01, ** *p* < 0.05, * *p* < 0.1.

## Data Availability

On-demand data availability.

## Appendix A. List of Eastern European Countries

Albania, Lithuania, the Czech Republic, Estonia, Hungary, Latvia, Slovakia, Slovenia, Moldova, Poland, Romania, Serbia, Bulgaria, Croatia, and Ukraine.

## Appendix B. Abbreviations

**Abbreviation**	**Explanation**
AMG	Augmented Mean Group
CCEMG	Common Correlated Effects Mean Group
CS-ARDL	Cross-Sectional Autoregressive Distributed Lag
CSD	Cross-Sectional Dependence
CO_2_	Carbon dioxide
GDP	Gross Domestic Product
EF	Ecological Footprint
EU	European Union
HO	Health Outcomes
IMR	Infant Mortality Rate
SH	Slope Heterogeneity
